# An improved method with high sensitivity and low background in detecting low β-galactosidase expression in mouse embryos

**DOI:** 10.1371/journal.pone.0176915

**Published:** 2017-05-05

**Authors:** Xiaopeng Shen, Wenjing Bao, Wei Yu, Rui Liang, Bao Nguyen, Yu Liu

**Affiliations:** 1 Department of Biology and Biochemistry, University of Houston, Houston, TX, United States of America; 2 The College of Life Science, Anhui Normal University, Anhui, China; 3 Department of Medicine, Liaoning University of Traditional Chinese Medicine, Shenyang, China; University of Minnesota Medical Center, UNITED STATES

## Abstract

*LacZ* is widely used as a reporter in studies of gene expression patterns. β-galactosidase, the product of *LacZ* gene, is usually detected by X-gal/FeCN staining. In X-gal/FeCN staining, β-galactosidase catalyzes X-gal to produce blue precipitates, which indicate the expression patterns of the gene of interest. A newer *LacZ* detection method using S-gal/TNBT is more sensitive but plagued by high background. Here, we describe an improved procedure that combines advantageous steps from the two methods. By comparing with X-gal/FeCN and S-gal/TNBT methods in detecting the expression patterns of *miR-322/503* and *miR-451* at a series of developmental stages, the improved method showed higher sensitivity and lower background. Thus, the improved method could be an alternative way of β-galactosidase staining in low gene expression situations.

## Introduction

*LacZ* is a common reporter gene used to study gene expression patterns [1, 2]. The protein product of *LacZ* is β-galactosidase, which catalyzes certain substrates to produce visible precipitates for ready detection. Often, *LacZ* is placed downstream of an endogenous promoter, in lieu of the endogenous open reading frame, to reveal the patterns of the endogenous gene expression. The most popular β-galactosidase substrate is X-gal. β-galactosidase catalyzes X-gal hydrolysis, giving rise to 5-bromo-4-chloro-3-hydroxyindole and galactose. 5-bromo-4-chloro-3-hydroxyindole is oxidized into a dimer that forms blue precipitates in the presence of potassium ferricyanide and potassium ferrocyanide [3, 4]. Although the X-gal/FeCN method exerts high specificity and low background, it fails when β-galactosidase expresses at low levels [5, 6]. A new S-gal/TNBT method was proposed as an alternative in detecting low β-galactosidase expressions [5]. S-gal is another chromogenic substrate of β-galactosidase, showing higher sensitivity than X-gal when used together with FeCN [7]. TNBT, unlike FeCN, forms formazan compounds in reducing conditions which appear dark-brown [8]. The S-gal/TNBT combination is much more sensitive than X-gal/FeCN, but has a severe overstaining problem. In the S-gal/TNBT protocol, the final chromogenic step has to be closely monitored and cannot be longer than 3 hours [5].

In our study of *miR-322/503*’s function in embryo development [9–11], we used β-galactosidase as a reporter in a “knockout first” mouse strain in which the *LacZ* expression cassette is inserted upstream of the *miR-322/503*-encoding sequence [12]. Initially, we used the X-gal/FeCN method to detect β-galactosidase activity. No signal was present at E8.5 and E9.5, and only weak signals were seen in E10.5 embryos. This was a disparity from other data. Chiefly, *miR-322/503* is enriched in Mesp1+ early mesoderm cells, suggesting that it is expressed as early as E6.25 [13–15]. Thus, we used the S-gal/TNBT method to explore the β-galactosidase reporter activity further. Though signals were readily detected in E8.5 embryos, they were obscured by high background. To optimize staining, we tried different combinations of fixation, wash and chromogenic staining, and found a method with high sensitivity and specificity. The new method works in a series of embryo stages in detecting low β-galactosidase expression.

## Material and methods

### Mouse strains and mating strategies

Animal Research (involved vertebrate animals, embryos or tissues): All work has been approved by the Institutional Animal Care and Use Committee (IACUC). Carbon dioxide inhalation was used for euthanasia.

**Fig 1:**
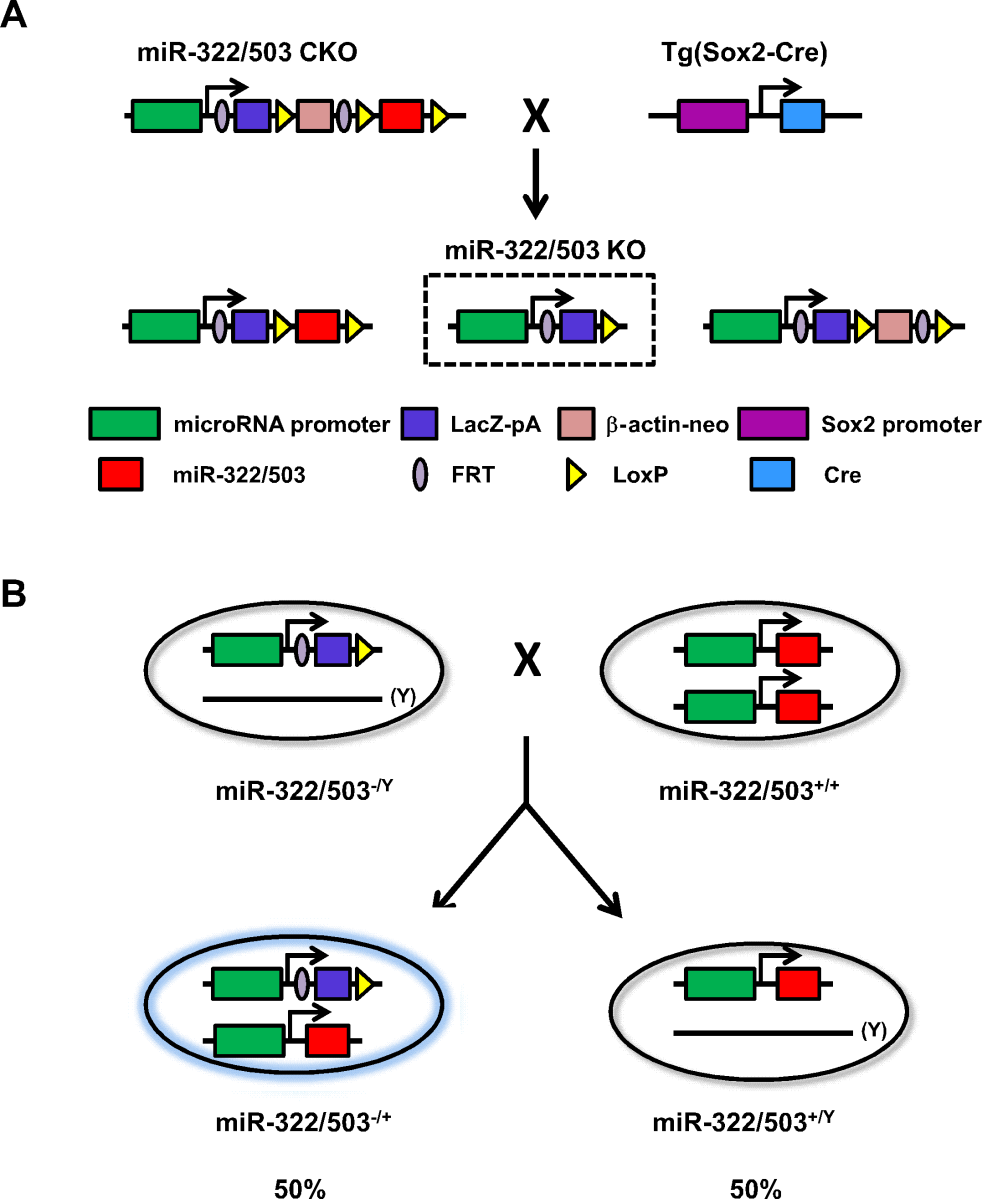
Schematic diagram of the *miR-322/503* knockout locus and the mating schedule. (**A**) *Mirc24*^*tm1Mtm*^/ Mmjax mice were crossed with Tg(*Sox2-Cre*) mice. Due to the presence of three LoxP sites, there were three possible alleles resulted from Cre-mediated recombination. We selected the one (dotted-box) with the *miR-322/503* stemloop ablated to carry out further mating and named it “*miR-322/503* KO”. (**B**) E8.5 embryos were produced from mating of male hemizygous knockout of *miR-322/503* (*miR-322/503*^-/Y^) with female wildtype SW mice. Half of the embryos are expected to be heterozygous knockout female mice (*miR-322/503*^-/+^) and also *LacZ* positive.

*Mirc24*^*tm1Mtm*^/ Mmjax, the “knockout first” mouse strain of *miR-322* and *miR-503*, was obtained from the Jackson Laboratory (Stock No: 017513) [12]. The male *Mirc24*^*tm1Mtm*^/ Mmjax mice were mated with the female Tg(*Sox2-Cre*) mice to generate female heterozygous knockout mice (*miR-322/503*^*-/+*^) of *miR-322* and *miR-503* (Fig 1). Next, *miR-322/503*^*-/+*^ mice were mated with wildtype male B6;129 mice to generate hemizygous male knockout mice (*miR-322/503*^*-/Y*^). Heterozygous female knockout embryos (*miR-322/503*^*-/+*^) were generated by mating the *miR-322/503*^*-/Y*^ mice with wildtype female Swiss (SW) mice. As the KO allele carries a *LacZ* expression cassette upstream of the coding sequence of *miR-322* and *miR-503* (“knockout first”), β-galactosidase serves as the reporter of *miR-322* and *miR-503* expression.

The *miR-451* promoter-*LacZ* is a transgenic mouse strain with the *LacZ* gene under the control of the 5-kb promoter of *miR-144/451* [16]. *miR-451* promoter-*LacZ* positive embryos were obtained by mating male transgenic *miR-451* promoter-*LacZ* mice with wildtype female SW mice.

The embryos were termed at embryo collection time. The start points of gestation were approximated to the middle time of the dark cycle just before the first observed vaginal plug. *LacZ* genotyping was determined by PCR primers: forward, 5’-CTCAAACTGGCAGATGCACGGT-3’; reverse, 5’-CGTTGCACCACAGATGAAACGC-3’.

### β-galactosidase staining

X-gal (5-Bromo-4-chloro-3-indoxyl-beta-D-galactopyranoside, Goldbio) was dissolved in dimethylformamide at 50 mg/ml. S-gal (6-chloro-3-indolyl-β-D-galactopyranoside, Sigma) was dissolved in DMSO at 50 mg/ml. The X-gal/FeCN and S-gal/TNBT methods include three steps each: fixation (F), wash (W), and chromogenic staining (S). In the X-gal/FeCN method, the embryos were fixed in 4% PFA for 15 minutes for E8.5 embryos and 30 minutes for E10.5 embryos (F1). Next, the embryos were washed three times with wash buffer (0.02% NP-40, 0.01% deoxycholate in PBS) for 15 minutes each (W1). Finally, the embryos were incubated with the staining solution (5 mM K_3_Fe(CN)_6_, 5 mM K_4_Fe(CN)_6_, 0.02% NP-40, 0.01% deoxycholate, 2 mM MgCl_2_, 5 mM EGTA, 1 mg/mL X-gal in PBS) in darkness at 37°C overnight. In the S-gal/TNBT method, the embryos were fixed in the fixation solution (0.2% glutaraldehyde, 2% formalin, 5 mM EGTA and 2 mM MgCl_2_ in 0.1 M phosphate buffer (pH 7.3)) for 15 minutes for E8.5 embryos and 30 minutes for E10.5 embryos (F2). Next, the embryos were washed three times with rinse solution (0.1% sodium deoxycholate, 0.2% IGEPAL, 2 mM MgCl_2_ in 0.1 M phosphate buffer (pH 7.3)) for 20 minutes each (W2). Finally, the embryos were incubated in staining solution (1 mg/ml Salmon gal and 0.4 mM TNBT in rinse solution) at 37°C (S2). S2 is monitored every 10 minutes and up to 3 hrs until specific staining appeared [17]. Both S1 and S2 steps were performed in a humidified chamber to prevent evaporation. We explored the different combinations of the three steps, as reported in Table 1. The established improved β-galactosidase staining method is shown in Table 2.

**Table 1:** Optimization of β-galactosidase staining.

	Fixation	Wash	Staining (1)	Staining (2)	Number of litters	Number of embryos	Number of positives	Expected positives
	F1	W1	S1	-	2	17	5	9
	F2	W2	S2	-	2	20	19	10
**A**	F2	W1	S1	-	3	27	0	14
**B**	F1	W2	S1	-	2	19	0	10
**C**	F1	W1	S2	-	3	24	24	12
**D**	F2	W2	S1	-	3	26	0	13
**E**	F2	W1	S2	-	3	24	24	12
**F**	F1	W2	S2	-	3	27	25	14
**G**	F1	W1	S1	S2	3	31	16	16
**H**	F2	W2	S2	S1	3	30	30	15
**I**	F1	W1	S1^S-gal^	-	2	18	10	9
**J**	F1	W1	S2^X-gal^	-	3	25	0	13

S1^S-gal^: X-gal was replaced with S-gal in S1 solution; S2^X-gal^: S-gal was replaced with X-gal in S2 solution.

**Table 2:** The established improved staining method.

	Reagents	Time and temperature
**Fixation**	4% PFA in PBS	15 minutes for E8.5 embryos, 30 minutes for E10.5 embryos; room temperature
**Wash**	0.02% NP-40, 0.01% deoxycholate in PBS	15 minutes, three times; room temperature
**Staining(1)**	5 mM K_3_Fe(CN)_6_, 5 mM K_4_Fe(CN)_6_, 0.02% NP40, 0.01% deoxycholate, 2 mM MgCl_2_, 5 mM EGTA, 1 mg/mL X-gal in PBS	Overnight in darkness; 37°C
**Staining(2)**	1 mg/ml Salmon gal, 0.4 mM TNBT, 0.1% sodium deoxycholate, 0.2% IGEPAL, 2 mM MgCl_2_ in 0.1 M phosphate buffer (pH 7.3)	Infrequently monitored until specific staining appeared; 37°C

## Results

### Comparing the two β-galactosidase staining methods

**Fig 2:**
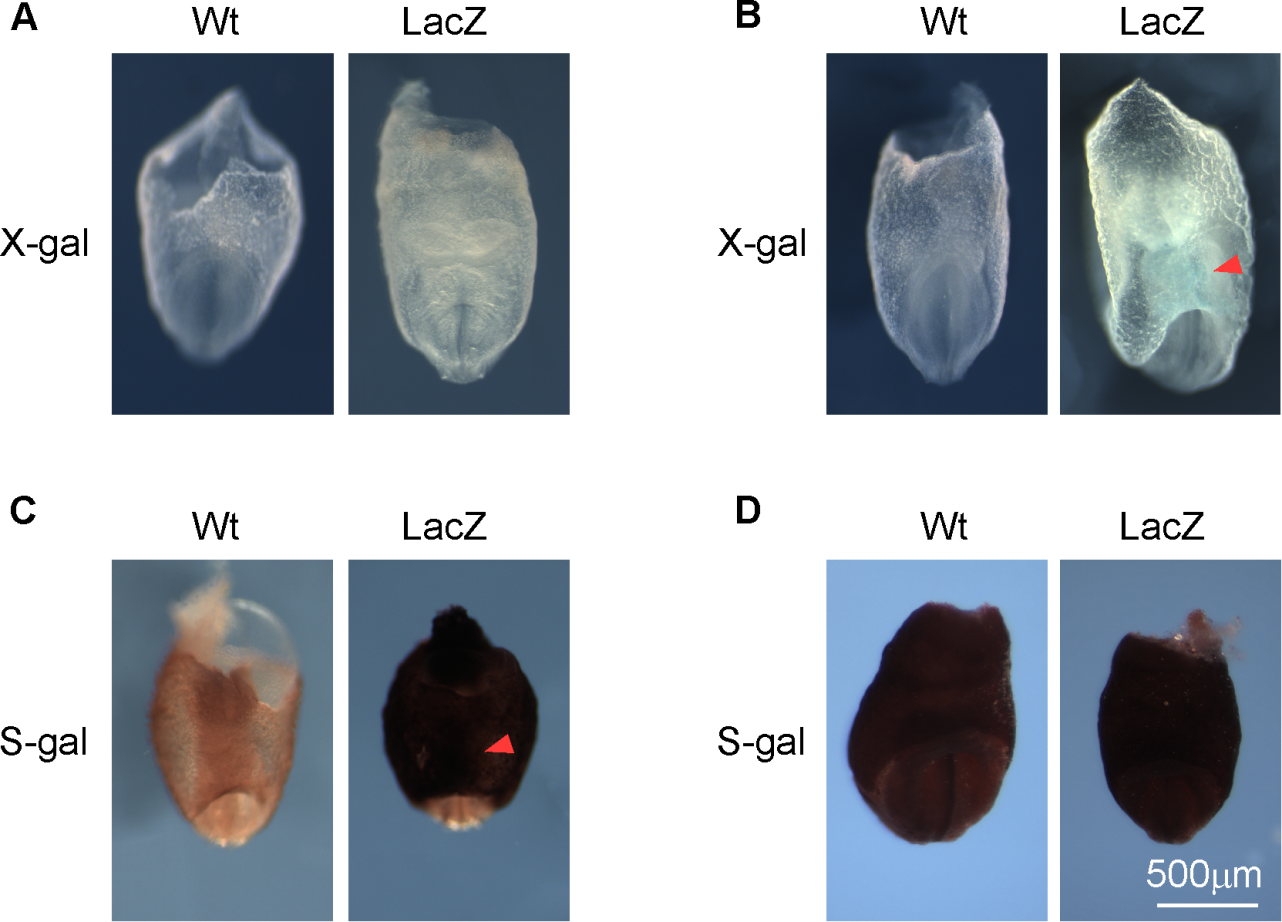
Comparing the X-gal/FeCN and S-gal/TNBT methods in staining *miR-322/503*-*LacZ* positive E8.5 embryos. (**A**) Wildtype and *LacZ* embryos stained with the X-gal/FeCN method overnight. (**B**) Wildtype and *LacZ* embryos stained with the S-gal/TNBT for 20 minutes. (**C**) Wildtype and *LacZ* embryos stained with the X-gal/FeCN method for two weeks. (**D**) Wildtype and *LacZ* embryos stained the S-gal/TNBT method for two weeks. The cardiac bulge is indicated with arrowheads.

We have established that the *miR-322/503* cluster plays an important role in early cardiac fate specification [9]. In order to appreciate the expression patterns of *miR-322/503* during embryogenesis, we employed a “knockout first” allele in which the *LacZ* reporter cassette was inserted upstream of the *miR-322* stemloop. Since *miR-322/503* was highly enriched in Mesp1+ cardiac mesoderm cells, we expected specific expression in cardiac related structures, such as the crescent. With the X-gal/FeCN assay, we could not detect any signals in E8.5 embryos, although RT-PCR results support that *miR-322/503* was expressed (Fig 2A and not shown). We extended the staining time of X-gal/FeCN to two weeks, and only to detect weak signals in the cardiac bulge (Fig 2C). We hence switched to a more sensitive assay using S-gal/TNBT. The S-gal/TNBT method led to strong signals in E8.5 embryos, highly enriched in yolk sac and the heart bulge, which matches Mesp1 expression patterns during embryogenesis, but the signals quickly merged into high background (Fig 2B and 2D). Our experience confirms the advantage and weakness of the two staining methods: the X-gal/FeCN method is highly specific but not sensitive, while the S-gal/TNBT method shows high sensitivity and also high background.

### Establishing an improved β-galactosidase staining method

**Fig 3:**
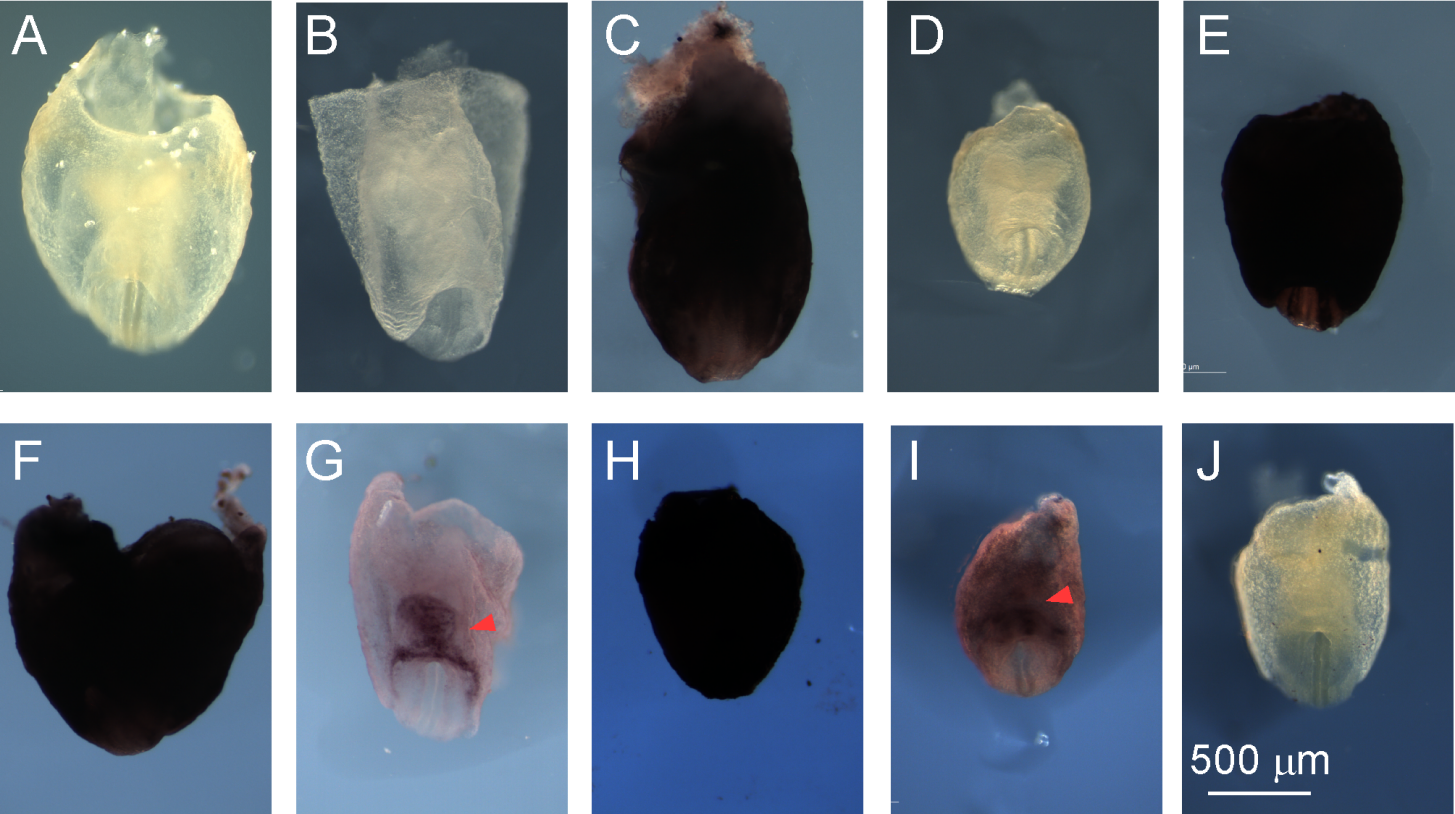
Optimization of β-galactosidase staining in E8.5 *miR-322/503*-*LacZ* embryos. (**A**)~(**J**) correspond to the group A~J in Table 1. “G” group showed the best staining outcome with the highest specificity and relatively low background. The cardiac bulge is indicated with arrowheads. Scale bar, 500 μm.

We would like to establish a β-galactosidase staining procedure that yields both high sensitivity and low background. A straightforward strategy was to combine the advantageous steps in the X-gal/FeCN and S-gal/TNBT methods. The staining procedure includes three steps: fixation, wash and chromogenic staining. For simple description, the three steps of the X-gal/FeCN method were named F1, W1 and S1. The three steps of the S-gal/TNBT method were named F2, W2 and S2. The steps from the two methods were hybridized, and the staining outcomes are shown in Table 1. None of the hybrid procedures (group A-F in Table 1) showed improvements over the original X-gal/FeCN or S-gal/TNBT methods (Fig 3A–3F). We observed that the procedures ending with S2 resulted in strong background while the procedures ending with S1 resulted in extremely low signals and background. Thus, the final chromogenic staining step was the source of difference in staining outcome between the X-gal/FeCN and S-gal/TNBT methods, not the fixation or washing steps. Next, we tried to add one additional chromogenic staining step to the original methods. When an additional S-gal/TNBT staining step (S2) was appended, the X-gal/FeCN method produced a strong and specific signal in the cardiac region of E8.5 embryos, without quickly developing high background (group G in Table 1, and Fig 3G). In contrast, when an additional X-gal/FeCN staining step was appended, the S-gal/TNBT method still produced strong background and the embryos became totally dark (group H in Table 1, and Fig 3H). These results suggest that either X-gal or other components of S1 help blocking nonspecific staining in the subsequent S-gal/TNBT chromogenic step.

We attempted to optimize the staining procedure in group G further, by experimenting a series of substrate/ buffer combinations. Our goal was to reduce the number of steps to 3. First, after the F1 and W1 steps, we used S-gal to replace X-gal in the S1 step but kept the other components of S1 (this new chromogenic staining step was designated S1^S-gal^). This scheme resulted in improvement when compared to the original X-gal/FeCN or S-gal/TNBT method, but still did not reproduce the sensitivity and specificity of group G (group I in Table 1 and Fig 3I). For comparison, after the F1 and W1 steps, we performed an S2 step in which we used X-gal to replace S-gal but kept the other components of S2 (this new chromogenic staining step was designated S2^X-gal^). This scheme did not show any improvement when compared to the original X-gal/FeCN method (group J in Table 1, and Fig 3J).

In summary, a new procedure comprising sequential X-gal/FeCN and S-gal/TNBT chromogenic staining had high specificity and low background in *miR-322/503*-*LacZ* staining in E8.5 embryos. The new procedure is described in Table 2.

### The improved method is highly reproducible

We looked to examine whether the improved β-galactosidase staining procedure is reproducible. We did statistical analysis of the stained embryos of all groups listed in Table 2, to determine if the staining results would match predicted Mendelian ratios. As neither *miR-322/503*^*-/Y*^ nor *miR-322/503*^*-/+*^ animals showed developmental defects, we bred *miR-322/503*^*-/Y*^ male with wildtype female SW mice, and predicted that the genotypes of E8.5 embryos obey the Mendelian ratio, which is 50% *LacZ* positive. The null hypothesis of chi-square test was positive and negative staining embryos were both 50%. The degree of freedom (df) in this test was 1. According to the standard chi-square value table, χ^2^ (P = 0.05, df = 1) = 3.84. When actual χ^2^ value is less than 3.84, we would not reject the null hypothesis that the staining results obey the Mendelian ratio. Finally, we found that among all test groups, group G produced staining results that most closely fit to the Mendelian ratio (“G” (16/31) (χ^2^ = 0.03), “J” (10/18) (χ^2^ = 0.22)). Thus, this new improved β-galactosidase staining method is highly reproducible. Comparing the staining and genotyping results showed that this improved method was accurate (16/16, 100%, staining positives/ genotyping positives).

### Testing the improved method in embryos at other embryonic stages

**Fig 4:**
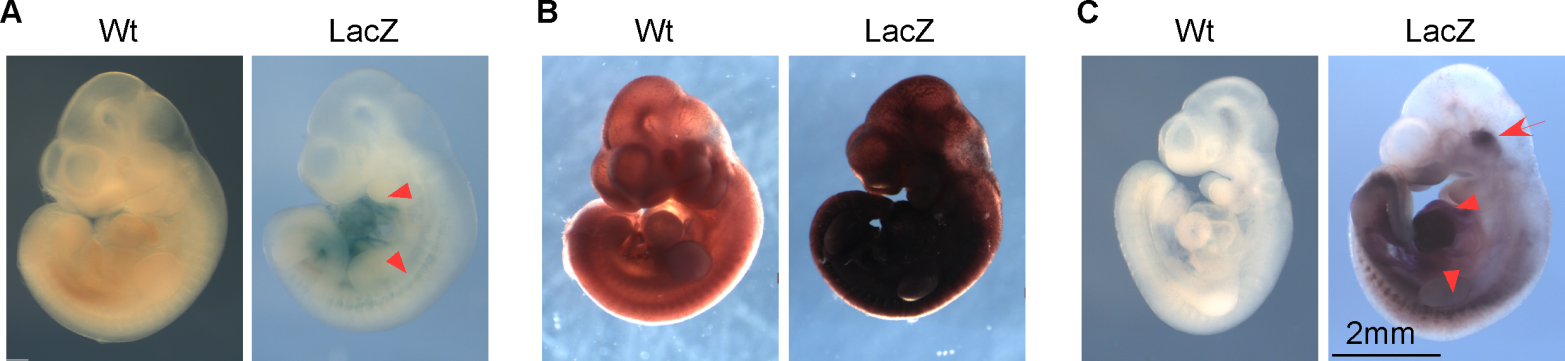
Verification of the improved method in E10.5 *miR-322/503*-*LacZ* embryos. (**A**) Wildtype and *LacZ* embryos stained with the X-gal/FeCN method. (**B**) Wildtype and *LacZ* embryos stained with the S-gal/TNBT method. (**C**) Wildtype and *LacZ* embryos stained with the improved method. The heart and somites are indicated with arrowheads. The signals around the cranial mesoderm and arches are indicated with an arrow.

Next, we asked if this improved method works in embryos of later stages. Our previous work established that *miR-322/503* specifically drove the cardiomyocyte and skeletal muscle lineages [9], therefore, we chose to test E10.5 embryos in which both the heart and somites are readily distinguishable. Positive staining was present using any of three methods (original X-gal/ FeCN, S-gal/TNBT and the improved methods). The X-gal/FeCN method produced specific light blue signals in the heart and somites, but only after overnight substrate incubation (Fig 4A). Within 30 minutes, the S-gal/TNBT method generated strong staining but also strong background (Fig 4B). The new procedure produced strong and specific staining in the heart and somites, and extended incubation was not necessary (Fig 4C). Additionally, positive signals were detected at the cranial level and arches. Our previous work has showed that *miR-322/503* was the most enriched miRNAs in Mesp1-lineage marked cells; and Mesp1-lineage marked cells populate the craniofacial mesoderm and arches. Thus, the positive signals are likely specific. In sum, the new method has higher sensitivity than X-gal/FeCN in later stage embryos.

**Fig 5:**
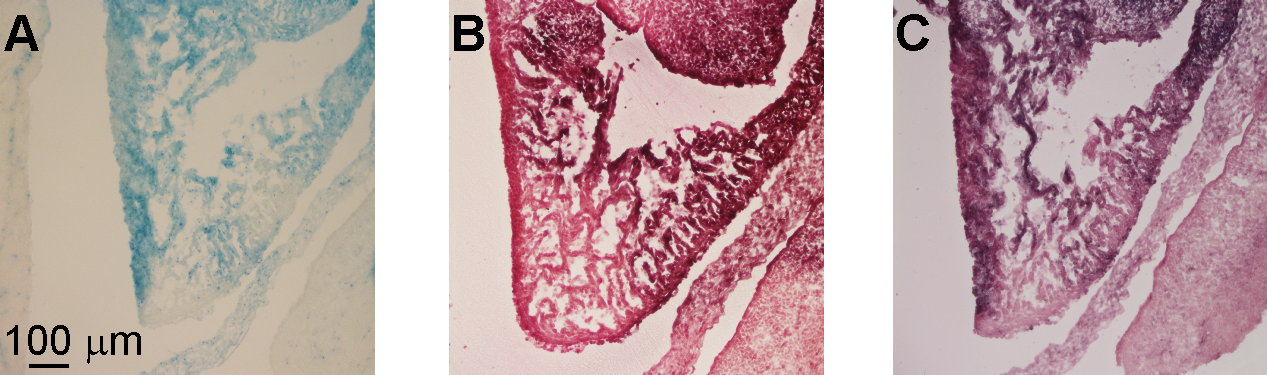
Verification of the improved method in E13.5 embryonic tissue sections. (**A**) Section stained with the X-gal/FeCN method. (**B**) Section stained with the S-gal/TNBT method. (**C**) Section stained with the improved method.

For older embryos, whole-mount β-galactosidase staining becomes impossible due to poor reagent penetration. Thus, we tested the performance of the improved method in tissue sections (Fig 5). In sagittal sections of E13.5 embryos, S-gal/TNBT produced strong signals, and the tissue resolution appeared to be superior than X-gal/FeCN staining. Overnight X-gal/FeCN staining produced the best signal/background ratio, but was not as sensitive as the other methods tested. Our new method produced similar results as S-gal/TNBT staining. The background noises seemed to be slightly less in the new method. The advantage of the new method in tissue sections is not as pronounced as in whole-mount embryos.

### The improved method is applicable for other *LacZ* reporters

**Fig 6:**
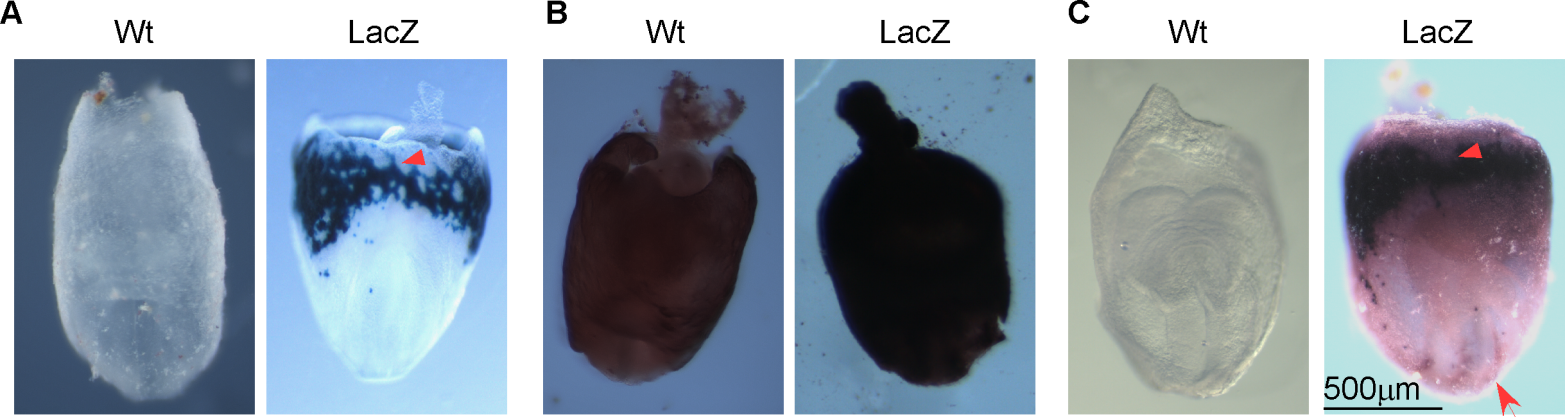
Verification of the improved method in E8.5 *miR-451*-*LacZ* embryos. The E8.5 *miR-451* promoter-*LacZ* embryos were generated from mating between transgenic male *miR-451* promoter-*LacZ* mice and wildtype female SW mice. (**A**) Wildtype and *LacZ* embryos stained with the X-gal/FeCN method. (**B**) Wildtype and *LacZ* embryos stained with the S-gal/TNBT method. (**C**) Wildtype and *LacZ* embryos stained with the improved method. The blood islands are indicated with arrowheads. Additional positive signals at notochord and endodermal lining of prospective midgut region are indicated with an arrow.

We used another *LacZ* reporter strain, *miR-451* promoter-*LacZ*, to test if the new method is broadly applicable. According to previous reports, *miR-451* is important for erythropoiesis under oxidative stress [18]. At E8.5, *miR-451* is localized in the yolk sac “blood islands”, which further differentiate into erythrocytes [16]. E8.5 *miR-451* promoter-*LacZ* positive embryos were produced from the mating between heterozygous male *miR-451* promoter-*LacZ* mice and wildtype SW female mice. Genotype-positive embryos were stained with the X-gal/FeCN method, the S-gal/TNBT method and the improved method. *miR-451* displayed clear “blood islands” staining with the X-gal/FeCN method, which suggests that *miR-451* has relatively high expression at E8.5 (Fig 6A). With S-gal/TNBT staining, we still saw darkened embryos with strong backgrounds (Fig 6B). When we tried to shorten incubation time to reduce backgrounds, we found that the backgrounds came out along with the staining in “blood islands”. When using the improved method, we detected specific and strong signals in “blood islands”, comparable to the results from the X-gal/FeCN method. Moreover, we detected signals in the notochord and endodermal lining of prospective midgut region (Fig 6C). In conclusion, the improved method showed not only specific staining similar as the staining from X-gal/FeCN method but also more staining details, which may indicate *miR-451*’s function in other parts of E8.5 mouse embryos.

## Discussion

β-galactosidase assay has been widely used in the detection of gene expression patterns. The most widely used X-gal/FeCN method was a reliable way in β-galactosidase staining with low background. However, X-gal/FeCN method was also relatively insensitive [19]. An alternative method, S-gal/TNBT, was proposed to detect patterns of low level gene expression, but frequent monitoring is required, otherwise high backgrounds rapidly develop [5]. In our study of *miR-322/503* expression pattern in E8.5 embryos, X-gal/FeCN showed extremely weak signals, while S-gal/TNBT displayed diffuse staining. We hence optimized the staining procedures, and developed a new method that allows rapid, sensitive, and specific β-galactosidase detection without frequent monitoring.

We tried to merge the advantages of X-gal/FeCN and S-gal/TNBT into one staining protocol. The most straightforward way was to recombine the staining steps of X-gal/FeCN and S-gal/TNBT. However, the direct recombination of staining steps did not exert improved outcomes. We then tried to add one additional chromogenic step to the original methods. The appendix of the S-gal/TNBT chromogenic step to the original X-gal/FeCN method yielded sensitive and specific staining. We further verified that this new protocol generated superior outcomes in embryos of more advanced stages, and in detection of β-galactosidase driven by an independent promoter.

The color reaction of X-gal/FeCN staining method is that 5-bromo-4-chloro-3-hydroxyindole, a product from the cleavage of X-gal by β-galactosidase, dimerizes and is oxidized with ferri- and ferrocyanide being electron acceptors. The final product was an insoluble blue compound. S-gal/TNBT method relied not only on cleavage and oxidation of S-gal but also the reduction of tetrazolium salts (for example, TNBT) to form colored formazan compounds. S-gal oxidation leads to pink or orange insoluble compounds but the process is slow. Formazan compounds rapidly react into other colored compounds. Therefore, the color displayed in S-gal/TNBT staining is mainly from TNBT reduction. TNBT introduces more backgrounds than ferri- and ferrocyanide since ferri- and ferrocyanide do not produce insoluble compounds. A possible explanation of our improvement is that X-gal/FeCN first makes the staining environment more oxidative, therefore, subsequent S-gal/TNBT would not as rapidly form colored formazan compounds. Additionally, X-gal/FeCN first reacts with nonspecific enzyme activities, allowing subsequent S-gal/TNBT to detect true β-galactosidase activities. In this way, the embryos stained with the improved method lead to high sensitivity and relatively low background.

In summary, we describe an improved procedure based on the existing X-gal/FeCN and S-gal/TNBT β-galactosidase assays. This procedure is particularly useful in detecting low β-galactosidase activities driven by weak promoters. In case that the conventional X-gal/FeCN method is proven not sufficiently sensitive, an additional S-gal/TNBT chromogenic step may reveal otherwise missed positive β-galactosidase signals. For strong promoters, the classic X-gal/FeCN method can produce excellent signal/noise ratios. In tissue sections, the three methods all produce satisfactory results, with the X-gal/FeCN method producing the best signal/noise ratios while the other two methods being faster and producing better tissue resolutions. Thus, our new method is a useful alternate to the existing β-galactosidase detection methods.

## Supporting information

S1 Fig Negative controls of β-galactosidase staining optimization in E8.5 *miR-322/503*-*LacZ* embryos. (**A**)~(**J**) correspond to the wildtype controls for group A~J in Table 1. Scale bar, 500 μm.
(JPG)
