# Supplementary figures and images for: An improved method with high sensitivity and low background in detecting low β-galactosidase expression in mouse embryos
Source: PLoS One. 2017 May 5;12(5):e0176915. doi: 10.1371/journal.pone.0176915 (PMC5419561; doi:10.1371/journal.pone.0176915)

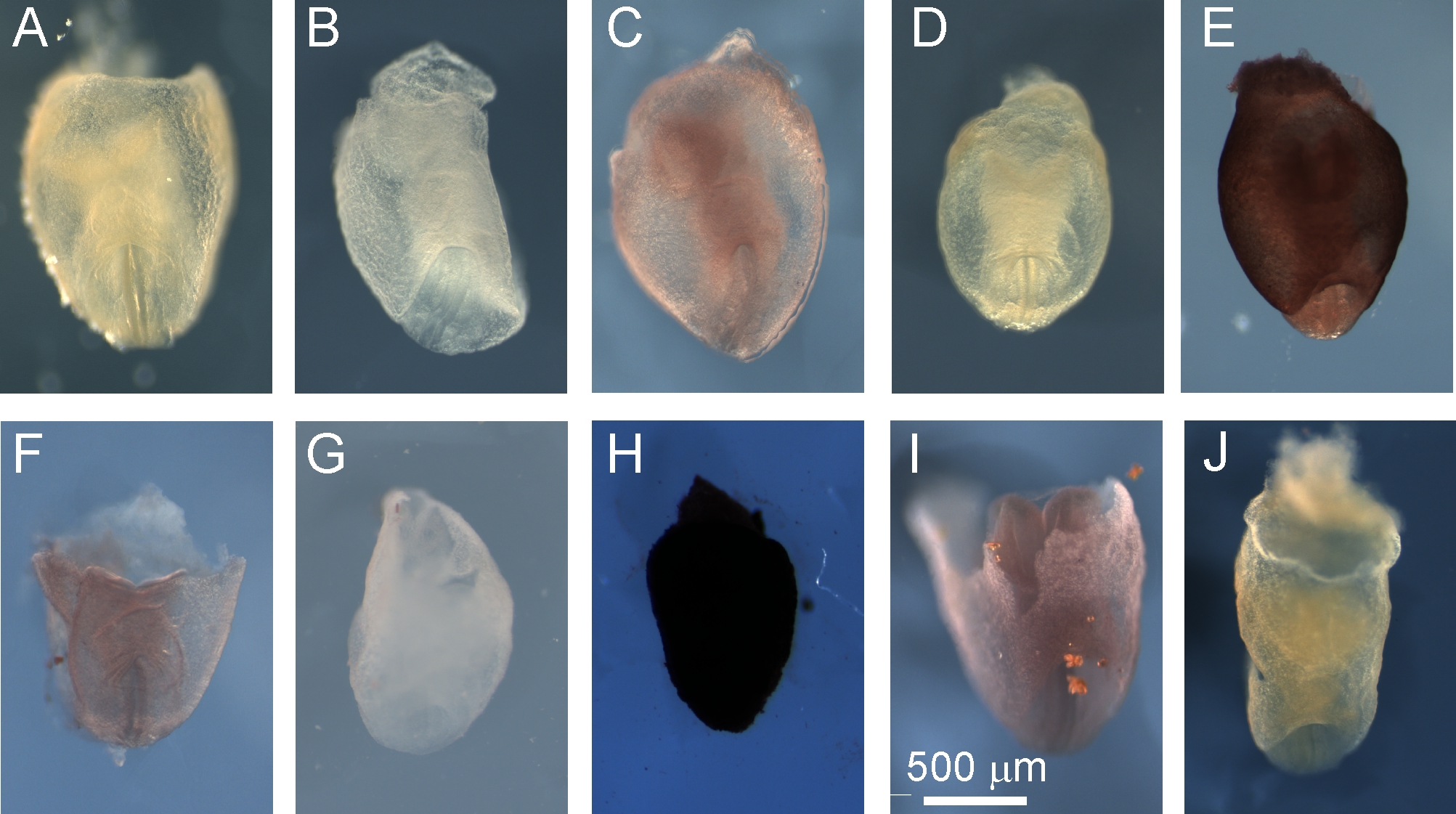

Supplement: S1 Fig — (A)~(J) correspond to the wildtype controls for group A~J in Table 1. Scale bar, 500 μm. (JPG) [file pone.0176915.s001.jpg]
